# Nutritional Health Perspective of Natto: A Critical Review

**DOI:** 10.1155/2022/5863887

**Published:** 2022-10-21

**Authors:** Muhammad Afzaal, Farhan Saeed, Fakhar Islam, Huda Ateeq, Aasma Asghar, Yasir Abbas Shah, Chigozie E. Ofoedu, James S. Chacha

**Affiliations:** ^1^Department of Food Science, Government College University, Faisalabad, Pakistan; ^2^Department of Home Economics, Government College University, Faisalabad, Pakistan; ^3^Department of Food Science and Technology, School of Engineering and Engineering Technology, Federal University of Technology, Owerri, Imo State, Nigeria; ^4^Department of Food Science and Agroprocessing, Sokoine University of Agriculture, P.O. Box 3006, Chuo Kikuu, Morogoro, Tanzania

## Abstract

Natto, a traditional soy food fermented by *Bacillus subtilis*, is made by steaming or cooking soaked soybean seeds, inoculating them with the bacteria, and then letting them sit for an incubation period. Natto soya has grown popular because of its nutritional importance and health advantages. As a result, farmers have more opportunities, thanks to the natto soybean market. For the natto soybean market to remain stable and grow, improved soybean cultivars with enhanced natto quality traits are essential. Natto's high-quality attributes are influenced by the bacteria strain, processing parameters, and soybean variety. Natto has a specific flavor and aroma with a slimy, sticky consistency. Natto possesses various therapeutic potentials and contains a range of essential nutrients and bioactive compounds, i.e., nattokinase, soybean isoflavone, *γ*-polyglutamic acid, vitamin K_2_, and biogenic amines. Bacterial species, processing conditions, and cultivars of soybean determine the quality characteristics of natto. Natto food is higher in menaquinone-7 and contains 100 times more menaquinone-7 than most cheeses. The present review highlights the production technology, microbiology, nutritional composition, and therapeutic potentials of natto.

## 1. Introduction

Natto is a fermented soybean food that was introduced thousands of years ago in North Japan [1]. There are three classes of natto, such as hamanatto, itohiki, and daitokuji natto. Itohiki natto is a kind of natto that has been inoculated with bacteria and cultured for 24 hours without the addition of salt. Daitokuji, or hamanatto, is prepared by injecting it with mold, raising it for 4–6 months, and adding salt to it [2]. Many states in Asia even have an equivalent product to natto, which includes the Philippines, Korea, Thailand, and China. Natto has 59% moisture, 16% protein, and 10% lipid in common; natto was produced from bristled soybeans and infused with *Grass bacillus* [3]. Soybean fermentation by *Grass bacillus* will create a mildewed flavor, an oily appearance, and a specific odor produced by sticky and viscous polymers [1]. Several compounds, including glutamic acid, amino acid, and fructan, are present in the sticky polymer. In Japan, natto was prepared with mustard seaweed, finely sliced onion, and a minor quantity of soy, so it was always presented with steaming rice [3]. Besides being a low-priced healthy food consumed with a pair of chopsticks, natto can also find application as a potential food mix in various food products. They should be coated with a white-colored slimy material that has a distinct flavor, a light yellow hue, and is able to produce a silky and sticky mass with a palatably soft texture. Furthermore, natto is used to flavor fish, meat, and vegetables [4]. Denaturation of soy proteins through heat, trypsin inhibitors, and bacterial-enzymatic protein degradation into simply digestible peptides boosts the nutritional value during the production process. After enough fermentation, the undamaged soybeans are covered in a white-colored sticky fluid and have a softer texture, a slimy apperance, and a distinct flavor [5]. Fermented natto products are also eaten without cooking and can be stored in cooler places or freezers, even within the supermarket, for selling and purchasing. Natto can be served with a couple of other food ingredients; for example, in Japanese homes, traditional natto is presented with seaweed, thinly chopped onion, mustard, and a small amount of condiment, and together with steamed rice, it is served as an entremet [6]. Natto can also be used for the preparation of meat, vegetable, and seafood dishes as a flavoring agent as well as an ingredient for the production of sauce [7]. Soybean fermentation produces proteolysis activity, which improves the taste and nutritional value by removing unwanted flavors [8]. Owing to the increase of isoflavone aglycone, the fermentation of soybean has an antidiabetic effect. To check the effects of fermentation time on total bioactive content and antioxidant activity, different time practices were made [9]. Additionally, different ratio practices have been done previosuly by incorporating *Saccharomyces cerevisiae*which affected the sensory characteristics and pH of black soybean natto. However, during the Taisho Period (1912–1926), scientists developed a method to synthesize *Bacillus natto* in the laboratory without the use of a straw. When placed in pots of cooked soybeans, the new laboratory-grown bacteria act as a dependable starter culture, allowing the production of natto [10]. Natto could subsequently be produced effectively by utilizing industrial-scale equipment such as big steaming/boiling caldrons. After adding the appropriate number of bacteria, the steamed beans might develop in any clean, non-corrosive container. Several biological events were reported during soybean fermentation by Hayashi et al. [11]. The amount and rate of the reactions and material formed are based on the situation of steaming, soaking, bacteria straining, and fermentation. Natto has an unusual flavor, smell, consistency, thickness, and quantity of mucus under different fusions of processing. Within this study, there are not even any small-level research laboratory techniques available to assess the soybean cultivar's acceptability for natto processing. Nowadays, black beans are solely used to make soy sauce, but black soybeans have been proven to be medicinally useful, with extracts used as an anti-inflammatory agent [12]. According to the most recent data, more than 700,000 tons of natto are produced each year. The present review highlights the therapeutic potential, production method, nutritional composition, and microbiology of natto in detail.

## 2. Production Technology of Natto

**Figure 1:**
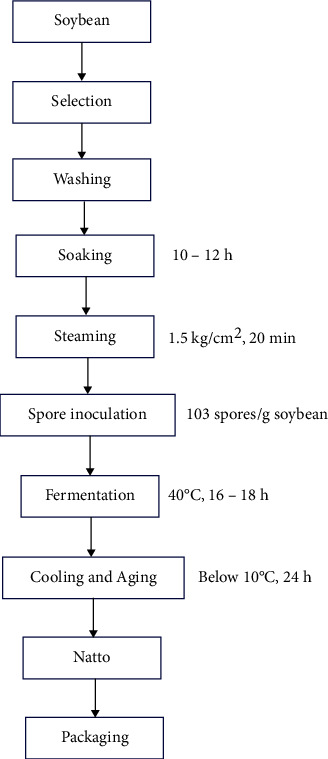
The mechanism of natto production is depicted in the flow sheet. Source: [14].

Natto is made from soybeans by the action of*Bacillus subtilis var. natto* (also known as *Bacillus natto*). Soybeans are a catch-all term for both black and green soybeans. Apart from the main components that separate soy proteins, fats, carbohydrates, cellulose, ash content, and moisture, there are also various trace elements and vitamins. *Bacillus natto* is a bacterium separated from traditional Japanese food, and its first type is similar to *Bacillus subtilis*, a subspecies of *Bacillus subtilis* [13]. Traditionally, it is wrapped in soybeans and stored in a warm area for 1–2 days. Computer network control provides a range of effective controls that may increase stable product quality, minimize utility consumption, and reduce production costs (Figure 1). They may also be used to manually participate in and monitor CR production, reduce quality risk, and reduce production costs.

## 3. Microflora and Microbiology

Natto production includes the following steps: washing and soaking whole grain soybeans overnight in hot water. The seeds are then cooked for 20–30 minutes at 0.98–1.47 Bar vapor pressure. Cooked beans are refrigerated to 45°C before being innoculated with a probiotic bacterial strain, and then natto is fermented for 18–20 hours at 40–45°C [15]. *B. subtilis* is a Gram-positive, fast-growing, aerobic bacterium with rod-shaped cells usually 2–6 *μ*m long and impartial below 1 *μ*m wide. The optimal temperature is at 30–35°C, which allows for twice as much time as 20 minutes. Under certain growth conditions, the cells form long chains that connect to unspecified septal wall components. Under starving conditions, cells can undergo a complicated two-cell division that results in endospore creation; this formation is discharged by lysis of the covering mother cell [16]. In other words, they can produce biofilms and “fruit bodies” containing grains. Isolated in the 1950s, tryptophan auxotroph was the most popular and studied type of *B. subtilis* 168. It was the first known gene to complete a genetic sequence, displaying a 4.2 Mbp chromosome containing 4100 genes. With a series of updates, the *B. subtilis* genome still has one of the best annotations. However, recently, “SubtiWiki” (a complete database) provides an easy-to-use and authentic configuration for the latest data (Table 1). Nicolas' work has resulted in a comprehensive data set for writing values, facilitators, and RNA controls on the website [18]. A complete list of key genes has been found in a number of global projects, recently identifying genes (the 257 genes) needed for *Lactobacillus* growth at 37°C ′(total genetics) of approximately 6250 genes and the “core genome” (genetic unit) of approximately 2500 genes. Considerable genetics involves a number of genes, about 300 genes were needed to build the endospore, as well as many prophages or fossils of the phage. The conclusions from the appearance of genetic matter are reconcilable with the idea that *B. subtilis* is modified for life in plants and the rhizosphere by Nicolas et al. [17].

## 4. Nutritional Composition

Natto provides 211 calories per 100 g. One serving contains 19 g of protein, 11 g of fat, and 13 g of sugar. The leftover carbohydrate complex contains 5.4 g of fiber and 4.9 g of sugar. Natto includes 1.6 g of saturated fat and provides 13.0 mg of vitamin C, 8.60 mg of iron, 729 mg of potassium, and 217 mg of calcium per 100 g. Therefore, natto is a food that falls under the category of “legumes and legume products.” Natto has a variety of carbohydrates. Each kind has its own range of advantages. One cup of natto contains around 6 grams of natural sugar. Another type of carbohydrate present in natto is fiber, Anderson et al. [19]. When you utilize a full cup of cooked food, you will obtain more than 9 grams of fiber. Adults should ingest 28 g of fiber per day, according to the USDA. Eating fiber not only helps digestion and exercise but it also has other health advantages, such as a lower risk of several cancers, obesity, heart disease, and diabetes [20]. One cup of natto has more than 19 g of fat. The majority of these lipids are polyunsaturated. Polyunsaturated fats can decrease LDL cholesterol and may reduce the risk of heart disease and stroke. Natto is a protein-rich food, so if we increase our plant-based protein intake by 34 grams while utilizing a big cup, natto contains an abundant source of micronutrients. The natto supplement gives 2.7 mg of manganese, a total of 130 to 134% of your recommended daily diet. It provides about 1 mg of copper (58% of your daily needs), 15 grams of iron (84% of your daily needs), 1276 mg of potassium (36%), 201 mg of magnesium (50%), 305 mg of phosphorus (30%), 5.3 mg of zinc (35%), and 15.4 mcg of selenium (22%). Food is high in ascorbic acid, which provides about 23 mg or about 38% of your daily requirements. The byproduct of the millet processing industry, millet bran, is rich in nutrients, particularly dietary fiber [21]. _2__6_, _1_Natto contains vitamins and other important compounds that helps to boost the immune system [22]. The food industry concentrates on using bioactive compounds because of the growing interest in doing so to maintain product quality and safety, as well as the benefits they have for human health and the environment [23]. Next, a discussion of applications and functionalization strategies for the administration of therapeutics via various delivery methods [24]. Natto has numerous health benefits, including the ability to control blood cholesterol levels, prevent arterial sclerosis, heart disease, and hypertension, promote bone growth, control the bacterial balance in the intestines, prevent diarrhea, enteritis, and constipation, improve immunity, fat reduction, beauty treatment, eye relief, and so on[25]. Finally, natto is frequently mentioned as one of the greatest sources of vitamin K, particularly vitamin K_2_. Vitamin K is used by the body to create bone and prevent blood clottingaccording to Anderson et al. [19]. Several studies on the health benefits of natto have been conducted; furthermore, in vivo and in vitro studies have demonstrated that serine proteases such as subtilisin and nattokinase have a profibrinolytic effect [26–28]. Mamiya and Nishimura [29] found that rats fed with natto had enhanced locomotor activity. Natto has some amazing benefits for bone development in menopausal women and postmenopausal bone loss prevention, which is most likely due to the presence of menaquinones or non-nato flavones in natto [30, 31].

## 5. Therapeutic Potential

### 5.1. Anticarcinogenic Activity

Natto has anticancer properties. A good example is miso soup, a well-known traditional Japanese dish that is prepared with soybeans as a major ingredient. This soup is basically made from the paste of soybeans that have been cooked with mold, yeast, and bacteria before being blended with water and salt. In order to prepare 200 ml of miso soup, commercially available natto in a quantity of about 50 g was added and cooked for 1 minute. All the volunteers ate miso soup daily at mealtime [32]. Human gastric adenocarcinoma cells were used to study the anticancer properties of chungkukjang (a Korean short-term fermented soy paste), and *Bacillus* strains from chungkukjang were isolated and identified. K-Chungkukjang (87%) demonstrated the strongest growth inhibitory effect at a concentration of 1 mg/mL, followed by H-chungkukjang (85%) and MC-chungkukjang (69%) (*P* < 0.05) reported by Seo et al. [33]. As per epidemiological studies, high levels of isoflavonoids are particularly related to a decreased colon cancer risk, while miso soup intake is linked to a lower risk of stomach cancer. Beans have been reported to hold large amounts of carcino-preventive agents. Bowel cancer was not that common in Japan, but it has now become more common among Japanese people due to the high-fat consumption in modern Japanese food, Adlercreutz [34].

### 5.2. Antibacterial Spectrum

**Figure 2:**
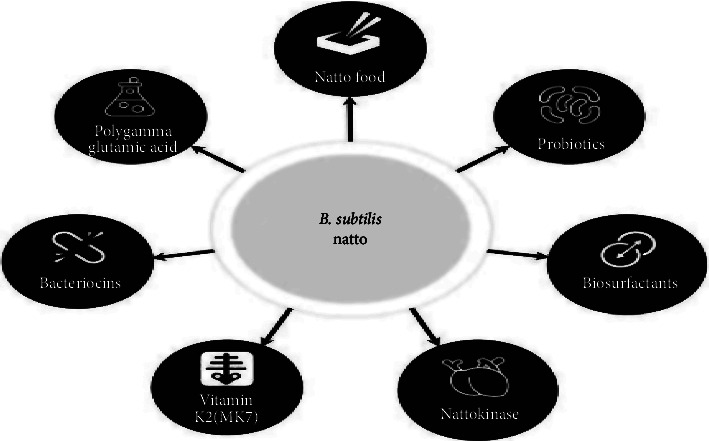
Potential effects of *Bacillus subtilis natto*. Source: [37, 38].

The microbial cytotoxicity of polyphenols may result from nonspecific interactions with polysaccharides, inhibition of proteolytic enzymes (peptidases), as well as other interactions that render bacterial adhesins deactivated [35]. Natto (*B. subtilis*) possesses antibacterial properties in terms of *Escherichia coli O157*. Growth of *E. coli O157* in culture and natto (*B. subtilis*) growth were both reduced. Natto promotes the growth of *Lactobacillus*, *Bacillus*, and *Streptococcus* while inhibiting the growth of *E. coli* in rat caeca reported by Sumi [36]. Natto's commercial diet contained a kind of *Bacillus* that might be beneficial as a biocontrol agent (Figure 2). Under optimal circumstances, natto displayed a substantially stronger inhibitory activity against *S. aureus*. Therefore, *Bacillus subtilis* has antibacterial action. Under well-designed circumstances, *B. subtilis* natto had the strongest activity in preventing *S. aureus*. Bactericidal action against *Helicobacter pylori* has been found in *Bacillus subtilis* natto. Because of the presence of dipicolinic acid, a small test of inhibitory concentration indicated that *B. subtilis natto* possessed antiplatelet aggregation and anti-*H. pylori* properties [39].

### 5.3. Effect on the Immune System

There has recently been a lot of excitement concerning the potential of immunotherapeutics. Tumor vaccines and immune checkpoint inhibitors are two immunotherapy strategies that have demonstrated a great promise in both clinical and preclinical trials [40]. Rivera-Patron et al. [41] investigated the effects of *Bacillus subtilis* natto on body function in dairy calves during the feeding period. *Bacillus subtilis* natto was mixed with milk and administered orally to calves. When the calves' initial diet reaches 2% of their body weight, they are fed. *Bacillus subtilis* natto enhanced working conditions by improving daily gain and nutritional efficiency, as well as increasing the weaning age of calves. In *Bacillus subtilis*, there were no increases in serum IgM, IgA, or IgE, but serum IgG was greater in natto-supplemented calves than in control calves studied by Rivera-Patron et al. [41].

### 5.4. Anti-Inflammatory and Hypocholesterolemic Effects

Soy protein with an eye or an eye alone has been demonstrated to decrease oxidative stress and have anti-inflammatory characteristics by decreasing nuclear factor-kappa B (NF–B) and preventing the release of chemical cytokines. The anticancer effect of lunasin (a peptide present in soybeans) comes from natto, which has antioxidative and anti-inflammatory properties. Lunasin, an antioxidant, was shown to slow down 2,20-azino-bis (3-ethylbenzothiazoline-6-sulfonic acid) diammonium salts and inhibit inflammatory cytokines (TNF- and IL-6) in RAW mouse 264.7 macrophages, Smithson et al. [42]. In general, soy isoflavones inhibit mTORC1 via the AKT pathway, which causes a decrease in lipogenesis and adipogenesis and an increase in lipolysis and oxidation in DIO male rats. This study shows soy isoflavones have amazing impacts on body weight and adiposity [43].

### 5.5. B-Galactosidase Activity

*Subtilis* bacterial cells (containing pNC61AV10 or pNZ2) were deposited in trimethoprim 10 milliliters of *Lactobacillus*Lactobacillus medium, and by the absorption rate of 600 nm, growth was examined. All the samples were mixed in, and the pellets were immersed in Z buffer (7 ml solution) (pH 7, 40 mM NaH_2_PO_4_–2H_2_0, 60 mM Na_2_HPO_4_–7H_2_0, 10 mM KCl, 1 mM phenyl methyl fluoro sulfate, 1 mM MgSO_4_–7H_2_0, and 2-mercaptoethanol). The cell suspension of 2.1 milliliters has three drops of toluene, and the solution was forcefully extracted for 9–10s. Then, to evaporate the toluene, the samples were stirred for 1 h at 37°C and collected at 28°C for 5 minutes. The reaction mixture was immediately stirred for 1 minute at 28°C, after adding 0.6 ml of orthonitrophenyl galactoside solution (pH 7, 4 mg of orthonitrophenyl galactoside per ml in 0.1 M sodium phosphate buffer). The reaction was stopped by adding 1 M Na_2_CO_3_ (1.5 ml) to the mixture. After the cells were discharged for 2 minutes, the absorption of clear supernatant (at 420 nm) was observed by Wang and Doi [44].

### 5.6. Gastrointestinal Proliferation

The use of extracts from certain *Bacillus* strains in natto, such as targeted microbial (direct-fed microbial), has been proven to have the ability to germinate and enter the digestive systems of diverse animals, such as poultry [45]. As a result, they are active and give a variety of nutritional advantages, such as the creation of exogenous enzymes such as cellulose, protease, phytase, lipase, keratinase, and xylanase, as well as other chemical compounds that improve the regulation of the body. In vitro*, Bacillus* spp. Xylanase was selected by using the in vitro digestive model. Cellulase production as DFM was then tested for digestion viscosity and *C. perfringens* to increase the diversity of chicken feed. The results of this study revealed that the use of less expensive grains (fermented soybean products) for poultry feed improved the digestion and activity level of birds found by Hendricks et al. [46].

### 5.7. Antidiabetic Effect

Hyperlipidemia and obesity are frequently linked with type II diabetes and insulin resistance, both of which lead to metabolic illness. Remarkably, fermented soybeans have antidiabetic and hypolipidemic properties in animals [47]. Soybean products that are fermented and contain soy protein, such as *Bacillus subtilis natto*, have been shown to be particularly helpful in reducing type 2 diabetes in humans. For six weeks, women aged 19–39 years were given a diet that included soy protein (20–30% other plant protein, 30–35% animal protein, and 30–35% soy protein). Jiang et al. [48] did an experiment and fed the rat soy protein-supplemented sucrose-rich foods. The results showed that the rat reduced cholesterol and hepatic triglyceride storage, steatosis, normal glucose-6-phosphate, and glycogen levels, and glucose transporter GLUT4 transplant. When this supplement is given for 4 weeks to diabetic Wister rats, it increases insulin sensitivity, insulin signaling, and pancreatic function studied by Kwon et al. [49].

### 5.8. Antiallergic Properties

An immune hypersensitivity disorder called allergy is caused by an allergy that enters the body through skin contact, injection, ingestion, and/or smell. These immune responses can develop into allergies, including inflammation such as atopic dermatitis, asthma, anaphylaxis, food allergies, and allergen rhinitis [50]. Lee et al. [51] found that natto has antiallergic effects on epidermis pigmentation, ear thinning, internal lymph nodes, and mast cell infiltration, among other things.

### 5.9. Antioxidative Properties

Many lifestyle-related disorders are induced by free radical oxidative damage to the living body. Reactive oxygen species and antioxidant mechanisms are out of balance, which leads to oxidative stress [52]. The study found that fermented-soy-products boosted antioxidant profile, total phenolic content, total flavonoid content, and isoflavones content. The variations in isoflavones might be attributed to -glycosidase activity [53]. Natto, or cooked beans, is a traditional Japanese meal that has been consumed for many years. Antioxidant activity has been demonstrated in the soluble components of natto water. Iwai et al. [54] have also reported that natto fractions suppress plasma low-density lipoproteins. LDL oxidation is recognized to play a role in the genesis and progression of arteriosclerosis. In this study, hypercholesterolemic mice fed a meal containing one or two natto components, a low molecular weight viscous or a soybean water extract, were found to have a considerable effect on LDL oxidation in vitro. To explore the inhibitory impact of natto fractions on LDL oxidation in vivo, lipid peroxidation in plasma and LDL were assessed following natto-treated mouse's plasma oxidation [54].

### 5.10. Effect on the Blood Pressure Level

Natto is a soybean derivative that is a popular traditional meal in Japan and is also used as a health supplement. Omura et al. [55] discovered that NKCP®, a natto supplement derived from the enzyme bacillopeptidase F, has antithrombotic, fibrinolytic, and antihypertensive properties. The utilization of dietary supplements present in traditional Japanese food provides further advantages in removing the independent symptoms of patients getting health treatment who have life-threatening diseases [56].

### 5.11. Protection against Apoptosis

Apoptosis is a hereditary cell death formation that plays an important function in cell number control. The diminished capacity to trigger apoptosis, which is coupled with changes in cell growth control systems, has a significant pathogenic feature in many forms of cancer [57]. The results from this study showed that *L. acidophilus* improved apoptosis in treated mice and reduced the severity of colorectal carcinogenesis. One of the most dangerous malignant epidermal cancers is melanoma. The natto, or soybeans fermented by *Bacillus subtilis natto*, was used to isolate natto freeze-drying extract (NFDE) and natto water extract (NWE), which were evaluated as potential antimelanoma agents (Table 2). Cell cytotoxicity tests showed that NFDE and NWE had significant, dose-dependent antimelanoma effects while having little effect on normal skin cells such as Hs68, HaCaT, and adipose tissue-derived stem cells (ADSCs) reported by Chou et al. [58].

## 6. Future Perspective

The development of food science in the near future probably depends on the continuation of active food science, an idea that was first introduced in Japan about 15 years ago. Japan, however, followed a unique path of progress in a product-driven environment rather than a science-driven science. In fact, the number of substances and products that have the potential to decrease the risk of disease rather than simply for health care has been investigated by their body-changing functions [59]. Some of them have been used in the manufacture of processed foods in accordance with the “defined health food use” officially defined by the new law. Probiotics have GRAS certification and are widely ingested across the world without any issues with safety. It has been demonstrated in a number of in vivo and in vitro studies. However, recent studies have revealed concerns about their safety and their capacity for immunocompetent individuals, Redman et al. [60]. However, probiotics are very healthy to promote health, especially in the prevention and treatment of diarrhea as well as *H. pylori* infection and maintaining intestinal homeostasis [61]. Their use with certain antibodies, especially for those who are severely ill, newborns, and the elderly, should be carefully monitored since reports of bacteremia in immune-prone patients treated with spores and other probiotics [62]. On the other hand, the importance of identification at the stage of severity is also significant to detect and eliminate any fundamental communication between probiotics and problems separated by allergies involved in the immune system. Therefore, it is essential to remember that clinical experiments involving these building materials should include a sufficient number of targeted individuals, including people with low immune systems. However, there is a dire need to conduct more clinical studies to investigate the therapeutic potential of natto.

## 7. Conclusion

Natto is a fermented soybean product that has a unique microbial profile and abundant bioactive compounds. The results of various studies conducted on natto suggest that natto has high probiotic potential. Significant research has been conducted to improve *B. subtilis natto* strains, with a focus on boosting the synthesis of useful chemicals such as nattokinase, PGA, and isoflavones, among others, hence improving the health benefits of natto soybeans. As a result, combining breeding soybean varieties with *B. subtilis natto* strains will allow for greater natto health promotion. There is strong evidence in the literature that *B. subtilis* natto has a high potential for producing probiotics for use in human food, and natto intake has been linked to health advantages such as a decreased incidence of certain illnesses and a lower risk of death.

## Figures and Tables

**Table 1:** Important microorganisms and enzymes used in food fermentation.

Microorganisms	Species	Active enzymes	References
Yeast	*Saccharomyces cerevisiae*	Amylase, proteases, maltase, dehydrogenase *β* glucosidase, and alcohol	Nicolas et al. [17]
Bacteria	*Bacillus subtilis*	Peptidase, hydrolases, proteases, amylase, and cellulase	Nicolas et al. [17]

**Table 2:** Lactic acid bacterial strains and their function.

Therapeutic property	Bacterial strains	Regulatory chemicals	References
Antioxidant damage DNA	*L. acidophilus* and *L. Casei*	↑ 5-fluorouracil	Elmore and Susan, [57].
Immune boosting	*L. acidophilus*	↑ DCs cell	Elmore and Susan, [57].
		↑ cytokines IL-12 and IL-10	

Epigenetics	Lipoteichoic acid and *L. acidophilus*	↑ expression of tumor suppressor genes	Elmore and Susan, [57].

## Data Availability

The data that support the findings of this study are available from the corresponding author upon request.
